# Decomposing rural–urban differences in successful aging among older Indian adults

**DOI:** 10.1038/s41598-022-09958-4

**Published:** 2022-04-19

**Authors:** T. Muhammad, Shobhit Srivastava, Babul Hossain, Ronak Paul, T. V. Sekher

**Affiliations:** International Institute for Population Sciences, Mumbai, Maharashtra 400088 India

**Keywords:** Geriatrics, Health policy

## Abstract

The modernization and shift towards urbanized lifestyles have triggered several diseases, and the context of aging varies in urban and rural settings in India. The study aimed to investigate the urban–rural differences in successful ageing among older adults in India and the contributing factors in those differences. The study utilizes data from nationally representative Longitudinal Ageing Study in India (LASI, 2017–18). The analytical sample size for the study was 31,464 older adults aged 60 years and above. Descriptive statistics and bivariate analysis were carried out to present the initial results. Multivariable logistic regression and decomposition analysis was used to find the associations between explanatory variables and successful aging and to identify the contributions of covariates that explain the rural–urban differences in successful ageing. A proportion of 32% and 24% of older adults from rural and urban areas were successful agers with an urban disadvantage. Urban-dwelling older adults had 0.67 times [95% confidence interval (CI): (0.64, 0.71)] lower unadjusted odds of successful ageing than rural older adults. Again, after adjusting for the effect of other explanatory variables, urban older adults had 0.92 times [CI: (0.87, 0.98)] lower odds of being successful agers than their rural counterparts. The major contributors to the rural–urban inequality in successful aging were differences in regional distribution (17% contribution), waist circumference (16%), working status (16%), body mass index (13%) and physical activity (8%) among rural and urban older adults. The urban disadvantage in aging successfully may reflect the higher prevalence of adverse lifestyle behaviours in urban dwellers and under-diagnosis and under-reporting of many diseases in rural areas, particularly non-communicable diseases, suggesting the need for further investigation.

## Introduction

With medical progress and improved lifestyle, populations in the developing countries have achieved increased life span in last few decades^1^. In addition, the effective public policies in the later ages have raised the share of the older populations in total population^1,2^. In India, the share of the population of age 60 years and above is predicted to increase from 8 per cent in 2011 to nearly 20 per cent in 2050^3^. As a result, with increasing ageing population, India and other developing countries confront different public health challenges such as high burden of morbidity and disability, increased demand for healthcare services, high health expenses and concerns related to the quality of life of the older people^4–6^. Thus, the understanding of the process and quality of aging becomes relevant and contemporary for many developing countries as well as for India^6,7^.

Rowe and Kahn (1997) argued that many of the age-related deteriorations that define the usual ageing process are associated with declining physical health status, lifestyle factors such as nutrition and diet, physical activity, behavioural and psychosocial factors^8^. However, Rowe and Kahn suggested that individuals involved in lifestyle adjustments and social engagement may avoid or diminish the effect of diseases and related physical health concerns and, consequently, enhance their likelihood of aging well^9^. Thus, Rowe and Kahn postulated the concept of successful ageing indicating less likely to suffer from chronic diseases and disability, high cognitive and physical functioning, and better social and productive engagement^10,11^.

Successful ageing has been found to be associated with socioeconomic status, adjustment in behavioural factors and range of physical and social activities, and emotional aspects^12–14^. For instance, adjustment in lifestyle such as avoiding smoking or drinking alcohol, regular meditation and dietary patterns can be crucial to achieve successful aging^12,13^. Better functional performance and higher physical activity are further found as important marker of successful aging^15^. Studies have also reported that the good social support can positively influence the successful aging^16,17^. Besides, social participation and engagement in different activities such as voluntary or charity work, involvement in political or community organization and frequencies of participation in such activity have positively associated with the successful aging^18,19^. Moreover, studies have reported that environmental factors and place of residence can be crucial for assessing successful aging^20–22^.

A considerable amount of literature suggests a difference in risk of many chronic diseases, disability, mental condition, and social engagement among older populations by their place of residence (urban and rural)^23,24^. It is mainly because of economic inequality, unequal accessibility to various type of health care services, and involvement gap in diverse social activities between urban and rural dweller elderly^25–27^. However, the findings regarding successful aging and its association with place of residence are inconsistent and mixed across the countries and study population^28^. For example, a China-based study found that, the urban dweller elderly had higher successful aging score than rural dweller elderly^28^. Whereas, a study reported that rural Korean and Japanese residents were more likely to be successful agers than their urban counterparts^29^.

In India, a major share of the older population is living in rural part of country^30^. It is evident that prevalence of different non-communicable diseases (NCDs) and risk factors for NCDs such as diabetics, hypertension, and obesity is higher in urban India^31,32^. Similarly, the gradual modernization and shift toward urbanized lifestyle further has triggered the lifestyle related diseases^33^. Even though, urban-residing older adults have more access to health services whereas, rural-dwelling older adults face difficulties availing the adequate healthcare service^34,35^. Due to all these, the context of aging may vary in urban and rural settings in India. While there are studies focusing on successful aging, limited studies have emphasized on urban–rural difference in successful aging in developing countries including India^36–38^. A better understanding of different factors associated with successful ageing such as individual, health-related or behavioural ones is essential in developing adequate health program and policies. Therefore, this cross-sectional study aimed to investigate the urban–rural differences in successful ageing among older adults in India and the contributing factors in those differences.

## Material and methods

### Data

This study utilized the data from India’s first nationally representative Longitudinal Ageing Study (LASI, 2017–18), which investigates the health, economic and social determinants and consequences of population ageing in India^39^. The representative sample included 72,250 individuals aged 45 years and above and their spouses across all states and union territories of India except Sikkim. The LASI adopts a multistage stratified area probability cluster sampling design to select the eventual observation units. Households with at least one member aged 45 and above were taken as the eventual observation unit. This study provides scientific evidence on demographics, household economic status, chronic health conditions, symptom-based health conditions, functional and mental health, biomarkers, health care utilization, work and employment. It enables the cross-state analyses and cross-national analyses of ageing, health, economic status and social behaviours and has been designed to evaluate the effect of changing policies and behavioural outcomes in India. Detailed information on the sampling frame is available in the LASI wave-1 Report and published elsewhere^39,40^. The effective sample size for the present study was 31,464 older adults aged 60 years and above.

### Variable description

#### Outcome variable

The outcome variable was dichotomous, i.e., successful ageing was coded as 0 “no” and 1 “yes”^41^. Successful ageing differs from region to region with no standard measurement, and the current study defined successful ageing following the modified version of the Rowe-Kahn’s model^41^, with the components of avoidance of disease and disability, maintenance of high physical and cognitive function and sustained engagement in social and productive activities. The components considered in the current study were (1) absence of chronic diseases (2) free from disability (3) high cognitive ability (4) free from depressive symptoms, and (5) active social engagement in life. The older adults satisfying all the above conditions were considered the successful ageing group^41^. The components in detail are as follow:

1. Absence of chronic diseases: Chronic diseases were assessed from the question “Have you been diagnosed with conditions listed below by a doctor?” The diseases were hypertension, chronic heart diseases, stroke, any chronic lung disease, diabetes, cancer or malignant tumour, any bone/joint disease, any neurological/psychiatric disease or high cholesterol^41^. Respondents were classified as having no chronic diseases if they reported none mentioned above.

2. Freedom from disability: Activities of Daily Living (ADL) is a term used to refer to normal daily self-care activities (such as movement in bed, changing position from sitting to standing, feeding, bathing, dressing, grooming, personal hygiene). The ability or inability to perform ADLs is used to measure a person’s functional status, especially in case of people with disabilities and older adults^42^. Respondents were classified as having no disability if they were ADL independent with no difficulty in performing any of the activity.

3. High cognitive ability: Cognitive function in the LASI survey was measured through five broad domains (memory, orientation, arithmetic function, executive function and object naming) adapted from the Mini-Mental State Examination (MMSE)^43^, and the cognitive module of the United States Health and Retirement Study (HRS) and its sister studies such as the China Health and Retirement Longitudinal Study (CHARLS), and the Mexican Health and Aging Study (MHAS)^44,45^. Memory was measured using immediate word recall and delayed word recall. Orientation was measured using time and place measures. The arithmetic function was measured through backward counting, a serial seven subtraction task and a task involving two computations^39,44^. Paper folding (folding a piece of paper according to instructions), pentagon drawing (drawing intersecting circles) and object naming methods were also followed to measure the cognitive functions among older adults^45^. A composite score of 0–43 was computed using the domain wise measures. The lowest 10th percentile measures poor cognitive functioning^39,46^. The older adults who did not fall into the category of lowest 10th percentile were considered as having a high cognitive ability.

4. Free from depressive symptoms: The probable major depression among older adults with symptoms of dysphoria was calculated using the Short Form Composite International Diagnostic Interview (CIDI-SF) with a score of 3 or more indicating major depressive disorder (MDD). The scale estimates probable psychiatric diagnosis of major depression and has been validated in field settings and widely used in population-based health surveys^47,48^. Older adults who did not fall into the “MDD” category were considered free from depressive symptoms.

5. Active social engagement: Respondents were said to be socially engaged if they participate in the following activities: eat out of the house (restaurant/ hotel); go to park/ beach for relaxing/ entertainment; play cards or indoor games; play outdoor games/ sports/ exercise/ jog/ yoga; visit relatives/ friends; attend cultural performances/ shows/ Cinema; attend religious functions/ events such as bhajan/ Satsang/ prayer; attend political/ community/ organization group meetings; read books/ newspapers/ magazines; watch television/ listen to the radio and use a computer for e-mail/ net surfing.

### Explanatory variables

#### Main group variable

Due to differences in lifestyles and disease patterns, aging differs in rural and urban areas. While defining the rural–urban group differences in the present study, place of residence was coded as rural and urban.

#### Individual factors

Age was coded as young old (60–69 years), old-old (70–79 years), and oldest-old (80 + years). Sex was categorized as male and female. Education was coded as no education/primary schooling not completed, primary completed, secondary completed, and higher and above. Marital status was categorized as currently married, widowed, and others (separated/never married/divorced). Working status was coded as currently working, retired/never worked, and currently not working. Living arrangement was coded as living alone, living with a spouse, living with children and living with others.

#### Obesity-related factors

Overweight/obesity was categorized as no and yes. The respondents having a body mass index of 25 and above were categorized as obese/overweight^49^. High-risk waist circumference was coded as no and yes. Male and females with waist circumferences of more than 102 cm and 88 cm respectively were considered high-risk waist circumference^50^. The high-risk waist-hip ratio was coded as no and yes. Male and females with a waist-hip ratio of more than or equal to 0.90 and 0.85 cm, respectively, were considered to have a high-risk waist-hip ratio^50^.

#### Behavioural factors

Tobacco and alcohol consumption was categorized as no and yes. Physical activity was coded as frequent (every day), rare (more than once a week, once a week, one to three times in a month), and never. The question through which physical activity was assessed was “How often do you take part in sports or vigorous activities, such as running or jogging, swimming, going to a health centre or gym, cycling, or digging with a spade or shovel, heavy lifting, chopping, farm work, fast bicycling, cycling with loads”?

#### Household factors

The monthly per-capita consumption expenditure (MPCE) quintile was assessed using household consumption data. Sets of 11 and 29 questions on the expenditures on food and non-food items, respectively, were used to canvas the sample households. Food expenditure was collected based on a reference period of seven days, and non-food expenditure was collected on reference periods of 30 days and 365 days. Food and non-food expenditures have been standardized to the 30-day reference period. The MPCE is computed and used as the summary measure of consumption. The MPCE variable was divided into five quintiles, i.e., from poorest to richest^39^. Religion was recoded into Hindu, Muslim, Christian, and Others. Caste was categorized as Scheduled Caste (SC), Scheduled Tribe (ST), Other Backward Class (OBC), and others. The SCs include a group of the socially segregated population and financially/economically by their low status as per Hindu caste hierarchy. The STs are among the most disadvantaged socioeconomic groups in India. The OBC is a group considered low in the traditional caste hierarchy that comes under intermediate categories in terms of socioeconomic status. The “other” caste category consists of people with higher social status who are not included in any of the above categories^51^. The region was coded as North, Central, East, Northeast, West, and South.

### Statistical approach

Descriptive and bivariate analyses were carried out to present the initial results. The proportion test evaluated the residential differentials and found the significance level^52^. Further, multivariable logistic regression analysis was used to find the associations of successful ageing with the place of residence and other explanatory variables. The estimates are presented in the form of crude odds ratio (COR) and adjusted odds ratio (AOR) with 95% confidence interval (CI). The adjusted odds estimates were controlled for individual, obesity-related, behavioural, and household factors.

Multivariate decomposition analysis was used to identify the contributions of covariates that explain the group differences to average predictions^53^. The decomposition analysis aimed to identify covariates that contributed to the difference in successful ageing by rural and urban residents. The multivariate decomposition analysis has two contribution effects: compositional differences (endowments) ‘E’ and the effects of characteristics that are the difference in the coefficients or behavioural change ‘C’ responses for the selected predictor variables^54^. The observed differences in successful ageing thus can be additively decomposed into a characteristics (or endowment) component and a coefficient (or effects of characteristics) component^55^. In the nonlinear model, the dependent variable is a function of a linear combination of predictors and regression coefficients:

$$Y=F \left(X\beta \right)=e(X\beta )/(1+ e\left(X\beta \right))$$

where Y denotes the n*1 dependent variable vector, X an n*K matrix of independent variables and $\beta$ a K*1 vector of coefficients.

The proportion difference in Y between rural A and urban B of successful ageing can be decomposed as:

$${Y}_{A}-{Y}_{B}=F\left({X}_{A}{\beta }_{A}\right)-F\left({X}_{B}{\beta }_{B}\right)$$

For the log odds of successful ageing, the proportion of the model is written as.
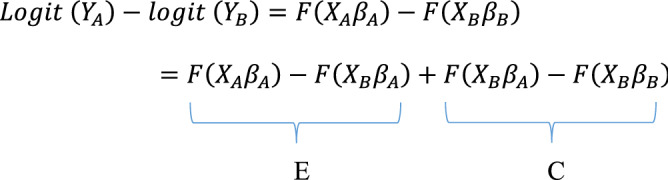


The component ‘E’ is the difference attributable to endowment change, usually called the explained component. The ‘C’ component is the difference attributable to coefficient (behavioural) change, usually called the unexplained component.

The model structure for the decomposition analysis was:

$$Logit \left(A\right)-Logit \left(B\right)=\left[{\beta }_{0A}-{\beta }_{0B}\right]+\sum {\beta }_{ijA}\left[{X}_{ijA}-{X}_{ijB}\right]+\sum {X}_{ijB}\left[{\beta }_{ijA}-{\beta }_{ijB}\right],$$

where

- ${\beta }_{0A}$ is the intercept in the regression equation for rural
- ${\beta }_{0B}$ is the intercept in the regression equation for urban
- ${\beta }_{ijA}$ is the coefficient of the ${j}^{th}$ category of the ${i}^{th}$ determinant for rural
- ${\beta }_{ijB}$ is the coefficient of the ${j}^{th}$ category of the ${i}^{th}$ determinant for urban
- ${X}_{ijA}$ is the proportion of the ${j}^{th}$ category of the ${i}^{th}$ determinant for rural
- ${X}_{ijB}$ is the proportion of the ${j}^{th}$ category of the ${i}^{th}$ determinant for urban

**Figure 1:**
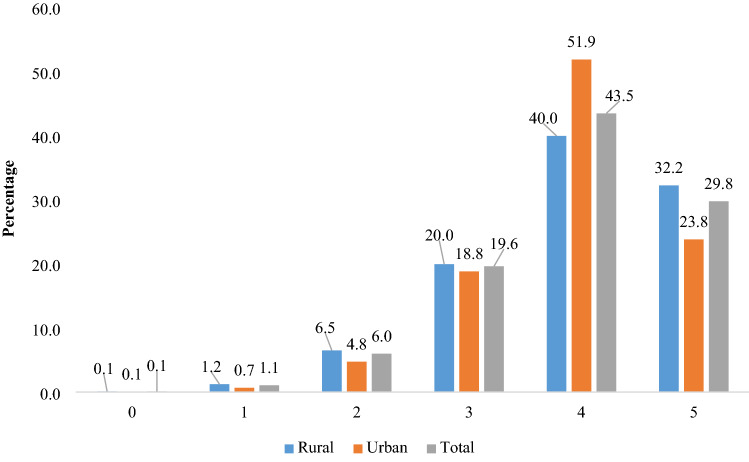
Distribution of participants by different levels of scoring for successful aging.

The command *mvdcmp* was used to perform multivariate decomposition analysis in STATA 14^56^ (Fig. 1).

### Ethics approval and consent to participate

The dataset used in the study is freely available in the public domain, and survey agencies that conducted the field survey for the data collection have collected prior informed consent from the respondents. The Indian Council of Medical Research (ICMR) extended the necessary guidance and ethical approval for conducting the LASI survey.

## Results

### Background characteristics

Table 1 shows the bio-demographic, behavioural, and household characteristics of 20,725 (66%) and 10,739 (44%) older adults residing in rural and urban communities, respectively. We observed that nearly six in ten older adults residing in urban and rural communities were in the young-old age group. Among older adults from the rural area, four in five had no formal schooling, one in five was currently not working, and one in four was living alone or with their spouse. Equivalently, one in two, one in three and one in five older adults from urban areas had no formal schooling, were not working and were either living alone or with their spouse, respectively. While 14% of older adults from rural areas were overweight or obese, and 66% never had physical activity, the same was higher in adults from urban areas (33% were overweight or obese; 77% never had physical activity).

**Table 1:** Socioeconomic profile of older adults in India, 2017–18.

Background characteristics	Rural		Urban	
	Sample	Percentage	Sample	Percentage
**Individual factors**				
**Age**				
Young-old	12,139	58.6	6,268	58.4
Old-old	6,169	29.8	3,354	31.2
Oldest-old	2,417	11.7	1,117	10.4
**Sex**				
Male	10,045	48.5	4,835	45.0
Female	10,680	51.5	5,904	55.0
**Education**				
Not educated/primary not completed	15,986	77.1	4,937	46.0
Primary	2,069	10.0	1,511	14.1
Secondary	1,988	9.6	2,598	24.2
Higher	682	3.3	1,693	15.8
**Working status**				
Currently working	7,341	35.4	2,106	19.6
Retired/never worked	8,774	42.3	4,719	43.9
Currently not working	4,610	22.2	3,913	36.4
**Marital status**				
Currently married	13,017	62.8	6,315	58.8
Widowed	7,280	35.1	4,162	38.8
Others	427	2.1	262	2.4
**Living arrangement**				
Living alone	1,311	6.3	444	4.1
Living with spouse	4,455	21.5	1,883	17.5
Living with children and spouse	13,708	66.1	7,873	73.3
Living with others	1,251	6.0	539	5.0
**Obesity-related factors**				
**Obese/overweight**				
No	17,863	86.2	7,160	66.7
Yes	2,862	13.8	3,579	33.3
**High-risk waist circumference**				
No	17,536	84.6	7,069	65.8
Yes	3,189	15.4	3,670	34.2
**High-risk waist-hip ratio**				
No	6,994	33.8	3,016	28.1
Yes	13,731	66.3	7,723	71.9
**Behavioural factors**				
**Tobacco consumption**				
No	11,353	54.8	7,886	73.4
Yes	9,372	45.2	2,853	26.6
**Alcohol consumption**				
No	17,465	84.3	9,523	88.7
Yes	3,260	15.7	1,216	11.3
**Physical activity**				
Frequent	3,980	19.2	1,610	15.0
Rare	3,101	15.0	813	7.6
Never	13,644	65.8	8,317	77.4
**Household factors**				
**MPCE quintile**				
Poorest	4,446	21.5	2,396	22.3
Poorer	4,608	22.2	2,197	20.5
Middle	4,375	21.1	2,207	20.6
Richer	3,932	19.0	2,117	19.7
Richest	3,364	16.2	1,822	17.0
**Religion**				
Hindu	17,309	83.5	8,497	79.1
Muslim	2,021	9.8	1,604	14.9
Christian	623	3.0	269	2.5
Others	772	3.7	369	3.4
**Caste**				
Scheduled Caste	4,572	22.1	1,220	11.4
Scheduled Tribe	2,125	10.3	325	3.0
Other Backward Class	9,213	44.5	5,056	47.1
Others	4,815	23.2	4,139	38.5
**Region**				
North	2,655	12.8	1,293	12.0
Central	4,920	23.7	1,533	14.3
East	5,678	27.4	1,573	14.7
Northeast	691	3.3	226	2.1
West	2,898	14.0	2,662	24.8
South	3,883	18.7	3,451	32.1
Total	20,725	100.0	10,739	100.0

### Bivariate analysis of successful aging by background characteristics

Table 2 shows the bivariate distribution of rural- and urban-dwelling older adults who experienced successful aging. About 32% and 24% of older adults from rural and urban areas were successful agers, respectively. We observed significant gender differences in successful aging, with 37% and 28% of men being successful agers in rural and urban areas compared to 28% and 21% of women in rural and urban areas. A higher proportion of older adults in rural areas experienced successful aging across all age groups than their urban-dwelling counterparts. Moreover, among older adults from rural areas who experienced successfully ageing, a higher proportion had higher education (36%), were currently working (45%), were currently married (36%), were not overweight or obese (34) and engaged in frequent physical activity (42%) in comparison to their urban resident counterparts (23%, 39%, 26%, 28% and 30% across the respective characteristics).

**Table 2:** Percentage of older adults with successful ageing among older adults by place of residence in India, 2017–18.

Background characteristics	Rural	Urban	Proportion test
	Row %	Row %	p-value
**Individual factors**			
**Age**			
Young-old	37.3	28.4	< 0.001
Old-old	27.3	18.8	< 0.001
Oldest-old	19.3	13.2	0.012
**Sex**			
Male	37.0	27.5	< 0.001
Female	27.7	20.8	< 0.001
**Education**			
Not educated/primary not completed	30.7	25.7	< 0.001
Primary	35.4	21.8	< 0.001
Secondary	39.7	21.7	< 0.001
Higher	36.0	23.3	< 0.001
**Working status**			
Currently working	44.9	38.6	< 0.001
Retired/never worked	24.6	21.8	< 0.001
Currently not working	26.6	18.3	< 0.001
**Marital status**			
Currently married	35.8	26.3	< 0.001
Widowed	25.6	19.8	< 0.001
Others	36.2	27.4	0.026
**Living arrangement**			
Living alone	24.0	20.0	0.041
Living with spouse	32.1	21.3	< 0.001
Living with children and spouse	33.8	24.7	< 0.001
Living with others	24.7	23.1	0.389
**Obesity-related factors**			
**Obese/overweight**			
No	34.0	27.8	< 0.001
Yes	21.1	15.8	< 0.001
**High risk waist circumference**			
No	34.4	29.0	< 0.001
Yes	20.5	13.9	< 0.001
**High risk waist-hip ratio**			
No	32.7	25.7	< 0.001
Yes	32.0	23.1	< 0.001
**Behavioural factors**			
**Tobacco consumption**			
No	30.0	22.2	< 0.001
Yes	35.0	28.3	< 0.001
**Alcohol consumption**			
No	31.3	23.0	< 0.001
Yes	37.0	29.9	< 0.001
**Physical activity**			
Frequent	42.3	30.0	< 0.001
Rare	42.3	28.5	< 0.001
Never	27.0	22.2	< 0.001
**Household factors**			
**MPCE quintile**			
Poorest	34.4	32.0	< 0.001
Poorer	34.6	25.5	< 0.001
Middle	33.6	25.7	< 0.001
Richer	31.7	17.1	< 0.001
Richest	25.0	16.4	< 0.001
**Religion**			
Hindu	33.0	24.7	< 0.001
Muslim	27.6	18.0	< 0.001
Christian	35.0	19.4	< 0.001
Others	25.3	31.6	0.008
**Caste**			
Scheduled Caste	31.1	28.9	< 0.001
Scheduled Tribe	41.4	36.7	< 0.001
Other Backward Class	32.1	23.2	< 0.001
Others	29.4	22.1	< 0.001
**Region**			
North	31.8	26.0	< 0.001
Central	38.9	32.4	< 0.001
East	33.6	24.1	0.002
Northeast	37.4	32.7	0.105
West	23.0	23.2	0.002
South	28.1	19.0	< 0.001
Total	32.2	23.8	0.000

Row %: Row percentage; p-value is based on two sample proportion tests.

### Multivariable logistic regression estimates of successful aging

Table 3 presents the multivariable logistic regression estimates for successful aging among older adults in India. From the unadjusted estimates, it was found that urban-dwelling older adults had 0.67 times [95% CI: (0.64, 0.71)] lower odds of experiencing successful aging than their rural counterparts. Again, after adjusting for the effect of other explanatory variables, urban-dwelling older adults had 0.92 times [CI: (0.87, 0.98)] lower odds of being successful agers than older adults from rural areas.

**Table 3:** Logistic regression estimates for successful ageing among older adults in India, 2017–18.

Background characteristics	COR	AOR
	(95% CI)	(95% CI)
**Place of residence**		
Rural		Ref
Urban	0.67* (0.64, 0.71)	0.92* (0.87, 0.98)
**Individual factors**		
**Age**		
Young-old	Ref	Ref
Old-old	0.66* (0.62, 0.7)	0.74* (0.7, 0.79)
Oldest-old	0.47* (0.43, 0.51)	0.57* (0.52, 0.63)
**Sex**		
Male	Ref	Ref
Female	0.66* (0.62, 0.69)	0.87* (0.81, 0.93)
**Education**		
Not educated/primary not completed	Ref	Ref
Primary	1 (0.93, 1.08)	0.99 (0.91, 1.08)
Secondary	1 (0.94, 1.08)	1.02 (0.94, 1.1)
Higher	0.84* (0.76, 0.92)	0.96 (0.87, 1.07)
**Working status**		
Currently working	2.5* (2.36, 2.65)	1.78* (1.66, 1.9)
Retired/never worked	Ref	Ref
Currently not working	0.92* (0.86, 0.98)	1.07 (0.99, 1.15)
**Marital status**		
Currently married	1.62* (1.54, 1.71)	1.22* (1.14, 1.31)
Widowed	Ref	Ref
Others	1.53* (1.31, 1.78)	1.3* (1.1, 1.54)
**Living arrangement**		
Living alone	1.16 (0.99, 1.35)	1.24* (1.05, 1.47)
Living with spouse	1.46* (1.29, 1.65)	1.12 (0.96, 1.29)
Living with children and spouse	1.45* (1.29, 1.62)	1.21* (1.06, 1.37)
Living with others	Ref	Ref
**Obesity-related factors**		
**Obese/overweight**		
No	1.96* (1.84, 2.09)	1.41* (1.29, 1.53)
Yes	Ref	Ref
**High risk waist circumference**		
No	2.19* (2.05, 2.33)	1.41* (1.29, 1.54)
Yes	Ref	Ref
**High risk waist-hip ratio**		
No	1.15* (1.09, 1.21)	0.95 (0.9, 1.01)
Yes	Ref	Ref
**Behavioural factors**		
**Tobacco consumption**		
No	0.74* (0.7, 0.78)	1 (0.94, 1.06)
Yes	Ref	Ref
**Alcohol consumption**		
No	0.74* (0.7, 0.79)	1.09* (1.02, 1.18)
Yes	Ref	Ref
**Physical activity**		
Frequent	2.1* (1.98, 2.23)	1.35* (1.26, 1.45)
Rare	1.96* (1.83, 2.1)	1.29* (1.2, 1.4)
Never	Ref	Ref
**Household factors**		
**MPCE quintile**		
Poorest	0.91* (0.85, 0.98)	0.93 (0.86, 1.01)
Poorer	0.85* (0.79, 0.92)	0.91* (0.84, 0.98)
Middle	0.72* (0.66, 0.77)	0.79* (0.72, 0.85)
Richer	0.6* (0.55, 0.64)	0.69* (0.63, 0.75)
Richest	Ref	Ref
**Religion**		
Hindu	Ref	Ref
Muslim	0.73* (0.68, 0.79)	0.76* (0.7, 0.83)
Christian	1.47* (1.37, 1.59)	1.05 (0.95, 1.17)
Others	0.88* (0.78, 0.98)	0.94 (0.83, 1.06)
**Caste**		
Scheduled Caste	1.29* (1.2, 1.39)	1 (0.92, 1.08)
Scheduled Tribe	2.08* (1.94, 2.24)	1.39* (1.27, 1.52)
Other Backward Class	1.19* (1.12, 1.27)	1.05 (0.98, 1.12)
Others	Ref	Ref
**Region**		
North	Ref	Ref
Central	1.54* (1.42, 1.68)	1.21* (1.1, 1.33)
East	1.18* (1.09, 1.28)	0.93 (0.85, 1.01)
Northeast	1.93* (1.77, 2.1)	1.32* (1.19, 1.47)
West	0.82* (0.75, 0.9)	0.68* (0.62, 0.75)
South	0.77* (0.72, 0.84)	0.72* (0.66, 0.78)

Ref: Reference; CI: Confidence Interval; COR: Crude odds ratio; AOR: Adjusted odds ratio; * if p-value < 0.05.

### Decomposition of rural–urban differences in successful aging

Table 4 shows the contribution of bio-demographic, behavioural and household characteristics to rural–urban inequality in successful aging among older Indian adults. We observed significant differentials in successful aging by place of residence, where 81% (Coef.: −0.016; SE: 0.005) of the inequality is attributable to the rural–urban differences in distribution of characteristics of older adults. Major contributors to the rural–urban gap in successful aging were differences in waist circumference (16% contribution), working status (16% contribution) and overweight/obesity status (13% contribution) among rural and urban older adults. Additionally, differences in physical activity among rural and urban older adults contributed to an 8% of the rural–urban gap in successful aging.

**Table 4:** Multivariate logistic regression decomposition estimates for rural–urban differentials in successful ageing among older adults in India, 2017–18.

Background characteristics	Due to difference in characteristics					Due to the difference in coefficients				
	Coef.	SE	p-value	% Contribution		Coef.	SE	p-value	% Contribution	
**Individual factors**										
**Age**										
Young-old	0.001	0.000	< 0.001	−0.7	−1.3	0.002	0.013	0.898	−2.1	1.2
Old-old	0.000	0.000	0.017	−0.5		−0.003	0.007	0.677	3.3	
Oldest-old										
**Sex**										
Male					0.2					−14.3
Female	0.000	0.000	0.454	0.2		0.012	0.009	0.171	−14.3	
**Education**										
Not educated/primary not completed					−0.3					2.9
Primary	−0.001	0.001	0.263	0.7		−0.002	0.002	0.192	3.0	
Secondary	0.001	0.001	0.362	−1.6		0.001	0.002	0.643	−1.0	
Higher	−0.001	0.002	0.794	0.6		−0.001	0.001	0.366	0.9	
**Working status**										
Currently working	−0.013	0.002	< 0.001	15.7	15.9	−0.004	0.006	0.537	4.7	−0.2
Retired/never worked	0.000	0.000	0.769	0.1		0.004	0.007	0.550	−4.9	
Currently not working										
**Marital status**										
Currently married	−0.001	0.000	0.002	1.0	0.2	0.000	0.010	0.987	−0.2	−0.2
Widowed										
Others	0.001	0.000	0.064	−0.8		0.000	0.001	0.989	0.0	
**Living arrangement**										
Living alone	0.000	0.000	0.274	0.5	−0.2	−0.001	0.002	0.631	1.2	57.7
Living with spouse	0.000	0.001	0.572	−0.6		−0.011	0.007	0.105	13.5	
Living with children and spouse	0.000	0.001	0.928	−0.1		−0.035	0.020	0.073	43.0	
Living with others										
**Obesity-related factors**										
**Obese/overweight**										
No	−0.010	0.002	< 0.001	12.8	12.8	−0.013	0.015	0.378	15.9	15.9
Yes										
**High-risk waist circumference**										
No	−0.013	0.002	< 0.001	16.3	16.3	0.017	0.015	0.263	−20.7	−20.7
Yes										
**High-risk waist-hip ratio**										
No	0.001	0.001	0.332	−1.0	−1.0	−0.001	0.004	0.886	0.7	0.7
Yes										
**Behavioural factors**										
**Tobacco consumption**										
No	−0.002	0.002	0.237	2.6	2.6	−0.012	0.008	0.101	15.2	15.2
Yes										
**Alcohol consumption**										
No	0.003	0.001	< 0.001	−3.5	−3.5	0.033	0.014	0.019	−40.5	−40.5
Yes										
**Physical activity status**										
Frequent	−0.003	0.001	< 0.001	3.6	8.0	−0.002	0.003	0.578	2.1	1.0
Rare	−0.004	0.001	< 0.001	4.3		0.001	0.003	0.729	−1.2	
Never										
**Household factors**										
**MPCE quintile**										
Poorest					−0.4					28.7
Poorer	0.000	0.000	0.009	0.1		−0.006	0.003	0.066	7.7	
Middle	0.000	0.000	0.027	−0.2		−0.003	0.004	0.365	3.9	
Richer	0.000	0.000	< 0.001	−0.4		−0.008	0.004	0.016	10.3	
Richest	0.000	0.000	< 0.001	0.1		−0.006	0.004	0.121	6.8	
**Religion**										
Hindu					4.7					4.2
Muslim	−0.004	0.001	< 0.001	5.1		−0.001	0.002	0.447	1.6	
Christian	0.000	0.000	0.676	−0.2		−0.002	0.003	0.371	2.8	
Others	0.000	0.000	0.661	−0.1		0.000	0.001	0.861	−0.3	
**Caste**										
Scheduled Caste	0.000	0.001	0.986	0.0	9.6	0.002	0.004	0.636	−2.0	−21.7
Scheduled Tribe	−0.008	0.002	< 0.001	9.6		0.007	0.004	0.084	−8.7	
Other Backward Class	0.000	0.000	0.070	0.0		0.009	0.006	0.105	−11.0	
Others										
**Region**										
North					17.4					8.4
Central	−0.003	0.001	0.064	3.2		−0.001	0.003	0.813	1.0	
East	0.005	0.001	< 0.001	−5.5		−0.011	0.004	0.009	13.6	
Northeast	−0.004	0.001	< 0.001	4.3		0.003	0.003	0.304	−4.2	
West	−0.004	0.001	0.002	4.9		0.005	0.002	0.018	−6.4	
South	−0.009	0.002	< 0.001	10.6		−0.004	0.004	0.333	4.4	
Constant						0.016	0.047	0.731	−19.5	−19.5
**Total**	−0.067	0.005	< 0.001	81.1	−0.016	0.007	0.034	18.9		

Coef.: Decomposition coefficients; SE: Standard error; % Contribution: Percentage contribution of each variable category to the overall rural–urban gap in successful aging among older Indian adults.

Furthermore, differences in tobacco consumption among rural- and urban-dwelling older adults were associated with a 3% gap in successful aging. Further, religion and caste-related differences among rural- and urban-dwelling older population contributed to a 5% and 10% of the rural–urban inequality in successful aging. Further, the regional gap among older adults contributed to 18% of the rural–urban gap in successful aging.

## Discussion

Using population-based, nationally-representative survey data, this is the first study to explore the rural–urban difference in successful aging and its contributing factors in India. Compared to the findings from other Asian countries, the successful aging scores of urban and rural older adults in this study were relatively higher, with 32.2% and 23.8% older adults meeting successful aging criteria in rural and urban areas respectively, probably due to the differences in definition and operationalization of successful aging in the study. For example, a comparative study between China and South Korea that excluded cognitive function from Rowe-Kahn’s model and added life satisfaction as a component of successful aging found 18.3% and 18.9% in China and 26.6% and 24.4% in Korea as successfully aging in rural and urban areas respectively^57^. Similarly, another study in China that included a different dimension of active engagement measure in their successful aging model reported 13.2% of the older adults as successful agers^58^. Using a multidimensional construct of successful aging encompassing absence of major chronic disease and difficulty in functioning, and maintenance of good psycho-cognitive function^59^, a Malaysian study found 13.8% of the participants as aging successfully^60^.

As documented in prior research, a considerable difference in demographic characteristics, socioeconomic status, and health care utilization exist between rural and urban populations in India^35,61,62^. In comparison to urban people, rural people are less educated, less healthy, more older, less likely to have income and employment^63^. Besides, there are considerably fewer physicians, hospitals and other healthcare services in rural communities than urban areas, and accessibility and affordability of healthcare are often limited by low income of people in these regions and inadequate transportation facilities^64,65^. Several studies in less-developed countries have also confirmed this discrepancy and observed a rural–urban gradient in healthcare utilization with rural residence of older people being negatively related to successful aging^57,58^. Nevertheless, the current analysis showed that a greater proportion of older adults residing in rural areas met the successful aging criteria than their urban counterparts.

Differences in successful aging among urban and rural populations with a rural residents’ disadvantage have been shown in multiple studies^28,66^. Although the healthcare system in India is poor especially the services provided to the residents of rural areas of the country, urban older people in India reported comparatively higher number of diseases^67–69^. Multiple studies have shown that rural elders to have fewer chronic conditions than urban elders^70,71^. However, as evidence suggests, such findings may be an artifact of under-diagnosis, under-reporting, under-ascertainment, and selective mortality^72,73^. It is also found that for diseases based on clinical assessment by the research team (hypertension and dementia), prevalence rates in urban and rural areas were similar^74^. Similarly, a study based on the data from the Study on Global Ageing and Adult Health (SAGE) revealed that the prevalence of several non-communicable diseases with standardized measures in urban people were higher than in rural people, possibly reflecting that urban dwellers might have better access to health care services for diagnosis and have better awareness of their health status^75^.

Also, the higher contribution of gender in rural–urban inequality in successful aging observed in our study may reflect the life course disadvantages of women in multiple dimensions of physical, functional and mental health. As suggested, if older women live longer with less probability of successful aging, the health problems may increase even further^74^, which in turn may result in lower score of successful aging in women in rural as well as urban areas.

Another possible explanation of the current finding is the noticeable differences in lifestyles between older persons in rural and urban residence. This is substantiated by the greater contribution of obesity/overweight, high-risk waist circumference and waist-hip ratio, smoking and alcohol drinking and physical activity in the rural urban inequality in successful aging observed in our study. A recent study also suggested a likely reverse causality of chronic conditions and physical inactivity, producing a vicious cycle between morbidity and unhealthy lifestyles^76^. Thus, physical activity and other health promotion programs should be implemented which could help preserve older people’s health and functions and reduce the risk of chronic diseases and ensure healthy aging. Meanwhile, participation in social activities which is a major component of successful aging should be encouraged in older populations especially in urban areas. For instance, cultural and physical activities and older people’s support groups could be encouraged to increase social support and psychological resilience, therefore promoting successful aging.

The higher contribution of household wealth quintile and caste status in rural–urban differences in successful aging suggests the inequity of rural and urban Indian older adults in their socioeconomic status and access to healthcare across the life course. The lower levels of socioeconomic status could also be associated with relatively lower social support which may result in lower psychological resilience in older people^77^. Consistent with our findings, a recent multi-country study reported that rural Korean residents were more likely to be successful agers than their urban counterparts which is possibly attributed to the rural to urban migration where older migrants could exhibit poor health status and suffer from financial burden of medical treatment^29^. Furthermore, a large portion of the urban Indian population is exposed to urban hazards such as pollution, traffic accidents and occupational injuries and related mortality^78–80^. For obtaining a comprehensive picture of the mechanisms playing in the rural–urban aspect of successful aging, the health status of older migrants from rural to urban areas in particular should also be evaluated in future studies.

The limitations of this study should be mentioned. First of all, it was a cross-sectional study, and no causal relationship could be determined. The prevalence of chronic conditions might be underestimated in rural India which could have resulted in considerable difference in successful aging in rural and urban areas. The data regarding the nutritional characteristics of the participants and their diet were not included while assessing successful aging. Similarly, the variables such as obesity-related measures and physical activity could be components of successful aging rather than factors that may explain it^9,81^. Such factors might play a major role in effective functioning in aging population which should be addressed by considering alternative definitions of successful aging in follow-up studies. The importance of specific socioeconomic and cultural context of India and similar countries for how aging is construed and later life is experienced needs to be explored. Future studies should also examine causal relationships in order to determine interventions that could improve successful aging in older Indian adults.

## Conclusion

We found that rural dwelling older Indian adults were more successfully aging than their urban counterparts. The study highlighted the major contribution of lifestyle factors, gender and socioeconomic status in the rural–urban differences in successful aging. The urban disadvantage in aging successfully may reflect the higher prevalence of adverse lifestyle behaviors in urban dwellers and under-diagnosis and under-reporting of many of the diseases in rural areas, particularly non-communicable diseases. Further studies are required to investigate the several mechanisms that play a major role in rural–urban differences in older individuals’ successful aging scores.

## Data Availability

The study utilizes a secondary source of data that is freely available in the public domain through a request. https://iipsindia.ac.in/sites/default/files/LASI_DataRequestForm_0.pdf.
