# Author Correction: Decomposing rural–urban differences in successful aging among older Indian adults

**DOI:** 10.1038/s41598-022-15751-0

**Published:** 2022-07-04

**Authors:** T. Muhammad, Shobhit Srivastava, Babul Hossain, Ronak Paul, T. V. Sekher

**Affiliations:** International Institute for Population Sciences, Mumbai, Maharashtra 400088 India

Correction to: *Scientific Reports* 10.1038/s41598-022-09958-4, published online 19 April 2022

T.V. Sekher was omitted from the author list in the original version of this Article.

The Author Contributions section now reads:

The concept was drafted by T.M. and S.S.; S.S. contributed to the analysis design, T.V.S. advised on the paper and assisted in paper conceptualization. T.M., S.S., B.H., R.P. and T.V.S. contributed to the comprehensive writing of the article. All authors read and approved the final manuscript.

The original Article has been corrected.

